# MRI severity of common extensor origin damage associated with prior glucocorticoid injection in chronic lateral epicondylitis: a retrospective multicenter analysis

**DOI:** 10.1186/s12891-026-10450-7

**Published:** 2026-09-05

**Authors:** Alexander-Stephan Henze, Larissa Eckl, Pavel Kadantsev, Thomas Ambacher, Philip Kuehn, Christian Schoch, Sebastian Siebenlist, Stephanie Geyer

**Affiliations:** 1 Division of Sports and Rehabilitation Medicine, Clinic for Internal Medicine II, Center of Medicine, University Hospital Ulm, Leimgrubenweg 14, 89075 Ulm, Germany; 2 Department of Musculoskeletal Surgery, University Hospital Ulm, Albert-Einstein-Allee 23, Ulm, 89081 Germany; 3 Department for Orthopaedic Sports Medicine, Klinikum rechts der Isar, Technical University of Munich, Ismaninger Straße 22, Munich, 81675 Germany; 4 SpOrt Medizin Stuttgart, Fritz-Walter-Weg 19, Stuttgart, 70372 Germany; 5 Nova clinic, Eichendorffweg 5, Biberach an der Riß, 88400 Germany; 6 Department for Orthopaedics, St. Vinzenz Klinik, Kirchenweg 15, Pfronten, 87459 Germany

**Keywords:** Tennis elbow, Elbow tendinopathy, Elbow injuries, Magnetic resonance imaging, Injections, Glucocorticoids, Conservative treatment

## Abstract

**Background:**

Lateral epicondylitis (LE) is a degenerative tendinopathy of the common extensor origin (CEO). Although local glucocorticoid injections are widely used for symptom relief, concerns exist regarding their potential adverse effects on tendon structure. This study aimed to investigate the association between prior glucocorticoid injection and magnetic resonance imaging (MRI)-based structural CEO damage in patients with chronic LE.

**Methods:**

In this retrospective, multicenter cross-sectional study, 147 patients with clinically diagnosed LE (symptom duration ≥ 6 months) who underwent elbow MRI between 2015 and 2023 were included. The primary exposure was prior local glucocorticoid injection at the CEO (yes/no and number of injections). Structural tendon damage was assessed using the Walz classification and quantitative lesion size diameters measured in coronal, axial, and sagittal MRI planes. Associations were analyzed using proportional odds ordinal logistic regression and generalized linear models, adjusted for age, sex, occupational workload, study center, and symptom duration.

**Results:**

Sixty-three patients (42.9%) had received at least one prior glucocorticoid injection. Prior injection was independently associated with more severe MRI-based tendon damage. Patients with prior injection had higher odds of being classified in a higher Walz grade compared with non-injected patients (adjusted odds ratio (OR) 2.78, 95% confidence interval (CI) 1.30–5.97; *p* = .008). Longer symptom duration was also associated with higher Walz grades (adjusted OR 1.02 per month, 95% CI 1.00–1.04; *p* = .031). Among injected patients, the number of injections was not significantly associated with Walz grade. Prior injection was further associated with larger lesion size diameters: +2.07 mm in the coronal plane (95% CI 0.80–3.34; *p* = .001), + 1.49 mm in the axial plane (95% CI 0.21 − 2.76; *p* = .022), and a 94% larger sagittal diameter (ratio 1.94, 95% CI 1.15–3.29; *p* = .013).

**Conclusions:**

Prior local glucocorticoid injection at the CEO was associated with more severe MRI-detected structural tendon abnormalities in patients with chronic LE. However, the retrospective cross-sectional design and potential for confounding by indication preclude causal attribution of these structural differences to glucocorticoid exposure. These findings provide imaging-based evidence supporting a cautious and selective approach to glucocorticoid injections.

**Supplementary Information:**

The online version contains supplementary material available at https://doi.org/10.1186/s12891-026-10450-7.

## Background

Lateral epicondylitis (LE), commonly referred to as tennis elbow, is a prevalent cause of lateral elbow pain and functional impairment, primarily affecting the common extensor origin (CEO) [1]. Once considered an inflammatory condition, LE is now widely recognized as a degenerative tendinopathy characterized by collagen disorganization, angiofibroblastic hyperplasia, neovascularization, and microtearing rather than acute inflammation [1–3]. The condition affects approximately 1–3% of the general population and is frequently associated with repetitive occupational or recreational loading of the wrist extensors [1, 4].

Magnetic resonance imaging (MRI) is an established modality for the evaluation of chronic LE, allowing detailed assessment of tendon signal alterations, partial- and full-thickness tears, and adjacent soft tissue pathology [5, 6]. MRI-based grading systems, such as the classification proposed by Walz et al., provide standardized assessment of structural CEO damage and have demonstrated correlations with clinical severity and surgical findings [7]. Importantly, imaging has gained increasing relevance not only for diagnosis but also for treatment stratification and outcome assessment in LE [8].

Management of LE is primarily non-surgical and may include patient education and activity modification, exercise-based rehabilitation and physiotherapy, physical modalities, and injection therapies, although the evidence supporting individual treatment modalities remains heterogeneous. Within this spectrum, local glucocorticoid injections have traditionally been used because of their rapid analgesic effects and widespread availability [1, 9]. However, high-quality evidence indicates that while glucocorticoid injections provide short-term pain relief, they are associated with inferior medium- and long-term outcomes, higher recurrence rates, and poorer functional recovery compared with placebo, physiotherapy, or alternative injectables [10, 11]. These limitations have prompted growing interest in alternative biologic or viscoelastic treatments, including platelet-rich plasma (PRP) and hyaluronic acid, which have demonstrated superior clinical outcomes, and, in some studies, favorable imaging-based tendon changes compared with corticosteroids [12–15].

Experimental and clinical studies further suggest that glucocorticoids may negatively affect tendon biology by suppressing tenocyte proliferation, impairing collagen synthesis, and reducing tendon mechanical strength, potentially accelerating degenerative changes and predisposing tendons to rupture [16, 17]. Despite these concerns, direct in vivo evidence linking prior corticosteroid injection to objectively quantified structural tendon damage at the CEO remains limited, particularly in patients with chronic LE assessed using standardized MRI criteria.

Therefore, the aim of this study was to investigate the association between prior local glucocorticoid injection at the CEO and MRI-based structural tendon damage in patients with chronic lateral epicondylitis. We hypothesized that a history of glucocorticoid injection would be associated with more severe MRI-detected CEO damage, assessed using both ordinal Walz grading and quantitative lesion size measurements.

## Methods

### Study aim, design, and setting

This retrospective, cross-sectional, observational multi-center study investigated the association between prior local glucocorticoid injection at the CEO and MRI-based structural CEO damage in patients with chronic LE. Patients were recruited from routine clinical visits at a shoulder and elbow specialist between 1 January 2015 and 31 May 2023 across three participating centers. MRI examinations had been performed as part of routine clinical care before inclusion in this retrospective analysis and were not obtained according to a study-specific imaging protocol or indication. The specific clinical indication for MRI was not consistently documented in a standardized manner and could therefore not be reliably reconstructed retrospectively.

The study was conducted in accordance with the most recent version of the Helsinki Declaration. Approval was obtained from the local ethics committees at all participating centers. Due to the retrospective study design and use of routinely collected clinical and MRI data, the requirement for informed consent was waived.

This study is reported in accordance with the *Strengthening the Reporting of Observational Studies in Epidemiology* (STROBE) guidelines [18]. A completed STROBE checklist is provided as Supplementary File 1.

### Study population

Patients were eligible for inclusion if they (i) had a clinical diagnosis of LE, (ii) reported a symptom duration of at least six months, and (iii) underwent MRI of the affected elbow including at least one fluid-sensitive coronal, axial, and sagittal sequence suitable for assessment of the CEO. Patients were excluded if (i) MRI data were incomplete, (ii) there was a history of injection with medications other than glucocorticoids or another invasive intervention at the CEO, or (iii) key exposure or outcome variables were missing. All eligible patients meeting the predefined inclusion and exclusion criteria during the study period were included; no a priori sample-size calculation was performed.

### Exposure variables

The primary exposure was prior local glucocorticoid injection at the CEO, assessed from medical records. Two exposure variables were defined: history of at least one glucocorticoid injection as a binary variable (yes/no), and total number of glucocorticoid injections administered prior to MRI. Detailed injection parameters, including glucocorticoid type and dose, injection technique (e.g., landmark- versus ultrasound-guided), and exact injection dates, were not consistently available. Consequently, the time interval between the last glucocorticoid injection and MRI could not be reliably reconstructed.

### MRI assessment and outcome variables

MRI assessment (1.5 T; at least one fluid-sensitive sequence in all three planes) was performed independently at each participating center by one experienced observer per center (orthopaedic surgeon with expertise in elbow MRI imaging). All observers were blinded to patients’ clinical data, including glucocorticoid injection history and symptom duration, to minimize assessment bias. Although inter-observer reliability was not formally assessed, the Walz classification has demonstrated good reproducibility in prior studies, and standardized assessment criteria were applied across centers.

#### Lesion size by Walz grade

MRI-based structural damage of the CEO was graded using the classification system proposed by Walz et al. [7], an ordinal scale ranging from grade 0 to grade 5, with higher grades indicating more severe tendon damage. Due to sparse observations in higher grades, Walz grades 3 to 5 were collapsed into a single category (≥ 3), resulting in a four-level ordinal primary outcome variable.

#### Lesion size by diameter measurements

In addition to ordinal grading, lesion size diameters of the CEO were measured in three orthogonal MRI planes (coronal, axial, and sagittal) in the image slice demonstrating the largest lesion extent. Diameters were recorded in millimeters (mm). A value of 0 mm indicated the absence of a measurable lesion in the respective plane, whereas values greater than 0 mm reflected the extent of CEO involvement. Accordingly, diameter measurements were treated as semi-continuous secondary outcome variables.

### Covariates

The following covariates were included a priori based on clinical relevance and prior literature: age (years), sex, symptom duration (months), study center (three centers), and occupational workload. Occupational workload was retrospectively classified by the study authors as low, moderate, or high based on the occupation documented in the electronic health record and expected work-related upper-extremity demands. Classification considered general physical workload, repetitive loading of the upper extremity, including repetitive forearm movements and prolonged mouse or keyboard use. As no validated occupational exposure instrument was available retrospectively, this approach represented a pragmatic categorization based on the available occupational information.

### Statistical analysis

Raw data were collected in Microsoft Excel for Mac (version 16.103) and imported into IBM SPSS Statistics (version 31.0.0.0) for analysis. GraphPad Prism10 (version 10.3.1) was used for graphical visualization. Descriptive statistics are presented as absolute and relative frequencies for nominal variables, medians with interquartile ranges (IQRs) for ordinal variables, and means with standard deviations (M ± SD) for continuous variables. Distributional assumptions for continuous variables were assessed using visual inspection (histograms and Q-Q plots) and the Shapiro-Wilk test.

The association between prior local glucocorticoid injection at the CEO and MRI-based structural damage graded according to Walz et al. was evaluated using proportional odds ordinal logistic regression. Injection history (at least one prior local glucocorticoid injection versus none) was included as the primary categorical exposure variable. Models were adjusted a priori for age, sex, occupational workload, symptom duration, and study center (fixed effects). Odds ratios (ORs) with 95% confidence intervals (95% CIs) represent common ORs across all cumulative thresholds of the collapsed ordinal Walz grading scale.

To investigate a potential dose-response relationship, a second proportional odds ordinal logistic regression model was fitted among patients with a history of glucocorticoid injection only, using the total number of injections as the exposure variable and adjusting for the same covariates. The dose-response analysis was restricted to injected patients to avoid structural collinearity between injection status and injection count. For both models, the proportional odds assumption was formally assessed using the test of parallel lines and was not violated.

The association between prior local glucocorticoid injection at the CEO and lesion size diameters at the CEO in coronal, axial, and sagittal MRI planes was assessed using generalized linear models. For coronal and axial diameters, models were specified with an identity link function and a robust (Huber-White sandwich) covariance matrix to account for potential heteroscedasticity. Models were adjusted for age, sex, occupational workload, symptom duration, and study center (fixed effects). Effect sizes of these generalized linear models are reported as adjusted mean differences with 95% CIs, expressed in millimeters. Exploratory analyses indicated a marked deviation from normality for sagittal lesion size diameters. To address this distributional characteristic, sagittal diameters were log-transformed after adding a small constant (+ 0.1) to accommodate present zero values. A log-linear generalized linear model with identity link was then fitted using the same covariate structure as described above. Model estimates were back-transformed, resulting in ratios. Effect sizes are reported as ratios with corresponding 95% CIs.

To facilitate comparability of effect sizes across secondary outcome scales, Cohen’s *d* was calculated as an additional effect for the generalized linear models. For linear models, Cohen’s *d* was derived directly from adjusted mean differences. For the log-linear model, Cohen’s *d* was approximated from log-transformed ratio estimates using the method described by Chinn, enabling consistent interpretation across generalized linear and non-linear models.

A two-sided *α* level of 0.05 was considered statistically significant for all analyses.

## Results

### Study population

A total of 147 patients with chronic LE (symptom duration ≥ 6 months) from three study centers were included in the final analysis. The mean age of the study population was 49.53 ± 9.12 years, and 75 patients (51.0%) were male. Prior local glucocorticoid injection at the CEO was documented in 63 patients (42.9%), whereas 84 patients (57.1%) had no history of injection. Among exposed patients, the mean number of injections was 1.71 ± 0.85 (range, 1–4). Baseline demographic and clinical characteristics, stratified by injection status, are summarized in Table 1.

**Table 1:** Baseline characteristics of the total cohort and stratified by glucocorticoid injection status

Characteristic	Total cohort *n* = 147	No history of a glucocorticoid injection *n* = 84	At least one prior glucocorticoid injection *n* = 63
Demographics			
Age, years	49.53 ± 9.12	50.18 ± 8.89	48.67 ± 9.42
Sex, male, *n* (%)	75 (51.0)	44 (52.4)	31 (49.2)
Occupational workload, *n* (%)			
Low	62 (42.2)	38 (45.2)	24 (38.1)
Moderate	62 (42.2)	33 (39.8)	29 (46.0)
High	23 (15.6)	13 (15.5)	10 (15.9)
Study center, *n* (%)			
1	57 (38.8)	28 (33.3)	29 (46.0)
2	32 (21.8)	11 (13.1)	21 (33.3)
3	58 (39.5)	45 (53.6)	13 (20.6)
Clinical characteristics			
Affected side, right, *n* (%)	84 (57.1)	46 (54.8)	38 (60.3)
Symptom duration, months	17.95 ± 17.74	17.94 ± 18.10	17.97 ± 17.40
Glucocorticoid injections, *n*	–	–	1.71 ± 0.85
MRI-based Walz grade, *n* (%)			
0	6 (4.1)	6 (7.1)	0 (0)
1	46 (31.3)	32 (38.1)	14 (22.2)
2	81 (55.1)	40 (47.6)	41 (65.1)
≥ 3	14 (9.6)	6 (7.1)	8 (12.7)
MRI-based lesion size			
Diameter coronal plane, mm	4.97 ± 3.64	4.12 ± 3.26	6.10 ± 3.84
Diameter axial plane, mm	5.95 ± 3.65	5.19 ± 3.74	6.95 ± 3.29
Diameter sagittal plane, mm	3.43 ± 2.83	2.6 ± 2.43	4.53 ± 2.95

Data are mean ± standard deviation, unless otherwise indicated

*Abbreviations*: *MRI* magnetic resonance imaging

### Walz-based MRI tendon damage

**Fig. 1:**
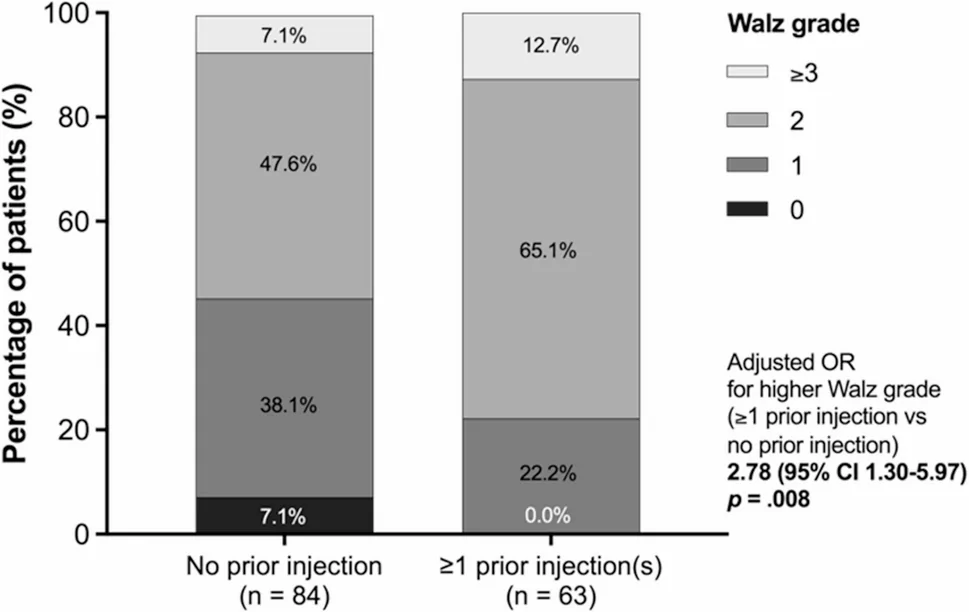
Distribution of patients with chronic lateral epicondylitis stratified by prior local glucocorticoid injection at the common extensor origin. Included were patients with a symptom duration ≥6 months who underwent elbow MRI at three participating study centers between 1 January 2015 and 31 May 2023, *n* = 147. Percentages are presented across Walz grades of MRI-based structural tendon damage. Percentages may not sum to 100% due to rounding. Due to sparse observations in higher grades, Walz grades 3 to 5 were combined into a single category (≥3), resulting in a four-level ordinal outcome. Abbreviations: MRI, magnetic resonance imaging

The overall median Walz grade was 2 (1–2). Higher Walz grades were more frequently observed in patients with a history of local glucocorticoid injection at the CEO (see Fig. 1).

In proportional odds ordinal logistic regression adjusted for age, sex, occupational workload, symptom duration, and study center, prior glucocorticoid injection was independently associated with more severe Walz-based CEO damage. The proportional odds assumption was satisfied (test of parallel lines, *p* = .778). Patients with a history of glucocorticoid injection had significantly higher odds of being classified in a higher Walz grade compared with non-injected patients (*β* = 1.024, SE = 0.389; adjusted OR = 2.78 (95% CI 1.30–5.97), *p* = .008; see Fig. 1). Longer symptom duration was also associated with higher Walz grades (*β* = 0.022, SE = 0.010; adjusted OR = 1.02 (95% CI 1.00–1.04), *p* = .031), whereas age and sex were not significantly associated with Walz-based tendon damage. Among patients with a history of local glucocorticoid injections, each additional injection was associated with a 7% increase in the odds of being classified in a higher Walz grade. However, this association was not statistically significant (adjusted OR = 1.07 (95% CI 0.55–2.09), *p* = .846).

### Diameter-based MRI tendon damage

Lesion signals were present in 83–88.4% of patients, depending on the MRI plane assessed. Across all planes, patients with prior glucocorticoid injection demonstrated larger lesion size diameters compared with non-injected patients. In adjusted generalized linear models with robust standard errors, prior glucocorticoid injection was associated with a 2.07 mm larger coronal lesion size diameter (95% CI 0.80–3.34, *p* = .001, *d* = 0.57) and a 1.49 mm larger axial lesion size diameter (95% CI 0.21–2.76, *p* = .022, *d* = 0.41). For sagittal lesion size diameters, which demonstrated substantial non-normality, a log-linear generalized linear model with robust standard errors was fitted. Prior glucocorticoid injection was associated with a 94% larger sagittal lesion size diameter on MRI (adjusted ratio 1.94, 95% CI 1.15–3.29, *p* = .013), corresponding to a standardized effect size of *d* = 0.37.

## Discussion

The most important finding of this multicenter retrospective study is that prior local glucocorticoid injection at the CEO is independently associated with more severe MRI-based structural tendon damage in patients with chronic lateral epicondylitis. Patients with a history of injection demonstrated significantly higher Walz grades and larger lesion size diameters across all MRI planes, even after adjustment for relevant demographic and clinical confounders. The observed effect sizes indicate quantitatively relevant structural differences, and the consistency of findings across ordinal Walz grading and lesion diameter measurements strengthens the observed association. However, because standardized concurrent clinical outcomes were unavailable, the clinical significance of these structural differences cannot be determined from the present study. Nevertheless, these findings provide objective imaging-based evidence that complements existing clinical outcome data and raise important concerns regarding the structural tendon effects of corticosteroid injections in chronic LE.

The present results are consistent with prior randomized trials demonstrating unfavorable long-term clinical outcomes following glucocorticoid injection compared with alternative treatments [10, 11]. More recent comparative studies have shown that non-steroidal injectables such as PRP or hyaluronic acid are associated with superior pain relief and functional outcomes and, in some cases, more favorable imaging-based tendon characteristics than glucocorticoids [12–15]. The present study extends this body of evidence by demonstrating an association between glucocorticoid exposure and objectively quantified structural tendon degeneration on MRI in a real-world cohort of patients with chronic LE.

The biological plausibility of these findings is well supported. Glucocorticoids have been shown to suppress tenocyte proliferation, induce apoptosis, impair collagen type I synthesis, and weaken extracellular matrix organization, ultimately reducing tendon mechanical strength [16, 17]. These effects may limit the tendon’s capacity to adapt to repetitive mechanical loading and promote progression from tendinosis to partial tearing. In this context, the observed increases in lesion size diameters and higher Walz grades are consistent with structural degenerative changes rather than transient inflammatory alterations.

From a clinical perspective, increasing structural damage of the CEO may have implications beyond isolated tendinopathy. The extensor–supinator mass plays an important role in dynamic stabilization of the lateral elbow, and advanced degeneration or tearing of the common extensor origin has been associated with an increased likelihood of concomitant posterolateral rotatory instability (PLRI) [19, 20]. In patients with chronic or refractory LE, higher-grade extensor tendon lesions are frequently accompanied by subtle insufficiency of the lateral ulnar collateral ligament, which may become clinically relevant as dynamic stabilization deteriorates [21, 22]. Given the association between glucocorticoid injections and increased structural CEO damage observed in the present study, structural changes of extensor tendon tissue in previously injected patients may contribute to structural conditions associated with PLRI, although this relationship was not directly assessed in the present study [7, 23].

Interestingly, although injection history was strongly associated with greater CEO damage, a statistically significant dose–response relationship with the number of injections could not be demonstrated. This may reflect limited statistical power within the injected subgroup, restricted variability in injection counts, or a non-linear relationship between injection count and structural abnormalities. The present data cannot distinguish among these explanations. Similar observations have been reported in other tendinopathies, where adverse tendon effects occurred after limited glucocorticoid exposure [24].

The consistency of associations across both ordinal grading and quantitative diameter measurements strengthens the robustness of our findings. While the Walz classification provides a clinically intuitive summary of tendon damage severity, diameter-based measurements offer a more granular assessment of lesion extent. The concordance of results across these complementary outcome measures suggests that the observed relationship is not dependent on a single imaging metric and supports the value of MRI as a tool for risk stratification in chronic LE [8].

Overall, these findings provide imaging-based evidence that supports cautious use of glucocorticoid injections in chronic LE and underscore the importance of considering structural tendon integrity in treatment decision-making.

### Strengths and limitations

Strengths of the present study are the multicenter design to improve generalizability, the blinded MRI assessment and dual outcome strategy, as well as robust regression analysis with covariate adjustment. However, several important limitations warrant consideration. Most importantly, the retrospective design precludes causal inference and is susceptible to confounding by indication. Standardized measures of symptom severity and baseline imaging before glucocorticoid injection were not consistently available in the retrospective records. Consequently, patients who received glucocorticoid injections may already have had more severe or structurally advanced disease before injection, and pre-existing differences in CEO damage between exposed and unexposed patients cannot be excluded. Adjustment for symptom duration and occupational workload accounts for some measurable differences between patients but cannot eliminate this residual confounding. Similarly, standardized clinical examination findings after injection were not consistently available, preventing direct assessment of the relationship between MRI-detected structural abnormalities and concurrent clinical symptoms or functional impairment. Furthermore, inclusion required the availability of an MRI suitable for CEO assessment. As the specific clinical indication for MRI could not be reliably reconstructed, selection or referral bias related to the decision to obtain MRI cannot be excluded. Inter-observer reliability of MRI assessment was not formally evaluated, although standardized criteria and only one experienced observer per center were used. Furthermore, details regarding glucocorticoid type, dosage, injection technique, and timing relative to MRI were unavailable and may have influenced tendon morphology. Finally, occupational workload was retrospectively classified from documented occupational information rather than assessed using a validated occupational exposure instrument, introducing the possibility of exposure misclassification. Nevertheless, generalizability may be limited to patients with chronic lateral epicondylitis undergoing MRI in specialized clinical settings.

## Conclusions

In conclusion, prior local glucocorticoid injection at the CEO was associated with more severe MRI-detected structural tendon abnormalities in patients with chronic LE. This association was consistently observed across ordinal Walz grading and quantitative lesion size measurements. However, the retrospective cross-sectional design, absence of pre-injection imaging and standardized clinical severity measures, and potential for confounding by indication preclude causal attribution of the observed structural differences to glucocorticoid exposure. These findings add imaging-based evidence supporting a cautious and selective approach to glucocorticoid injections in chronic LE and warrant prospective longitudinal studies incorporating baseline imaging and standardized clinical outcomes.

## Supplementary Information

Supplementary Material 1.

## Data Availability

The datasets generated and/or analyzed in the current study are not publicly available due to the anonymity of patients but are available from the corresponding author on reasonable request.

## Abbreviations

CEO: Common extensor origin
CI: Confidence interval
LE: Lateral epicondylitis
MRI: Magnetic resonance imaging
OR: Odds ratio
PLRI: Posterolateral rotational instability
PRP: Platelet-rich plasma
SD: Standard deviation
STROBE: Strengthening the reporting of observational studies in epidemiology
