# Supplementary material for: MRI severity of common extensor origin damage associated with prior glucocorticoid injection in chronic lateral epicondylitis: a retrospective multicenter analysis
Source: BMC Musculoskelet Disord. 2026 Sep 5;27:813. doi: 10.1186/s12891-026-10450-7 (PMC13629001; doi:10.1186/s12891-026-10450-7)
Supplement: Supplementary file 1 [file 12891_2026_10450_MOESM1_ESM.docx]

| **Section** | **Item** | **STROBE recommendation (abridged)** | **Reported on page / location** |
| --- | --- | --- | --- |
| Title and abstract | 1 | Indicate the study design in the title or abstract and provide an informative, balanced summary. | Title, p. 1; Abstract, pp. 2–3 |
| Introduction – Background/rationale | 2 | Explain the scientific background and rationale for the investigation. | Background, pp. 3–4 |
| Introduction – Objectives | 3 | State specific objectives and any prespecified hypotheses. | Background, p. 4 |
| Methods – Study design | 4 | Present key elements of the study design early in the manuscript. | Methods: Study aim, design, and setting, pp. 4–5 |
| Methods – Setting | 5 | Describe setting, locations, and relevant dates, including the study period. | Methods: Study aim, design, and setting, pp. 4–5 |
| Methods – Participants | 6 | Describe eligibility criteria and how participants were selected. | Methods: Study population, p. 5 |
| Methods – Variables | 7 | Clearly define outcomes, exposures, predictors, confounders, and relevant diagnostic criteria. | Methods: Exposure variables, MRI assessment and outcome variables, Covariates, pp. 5–7 |
| Methods – Data sources/measurement | 8 | For each variable, describe data sources and measurement/assessment methods; indicate comparability across centers where relevant. | Methods: Exposure variables, MRI assessment and outcome variables, Covariates, pp. 5–7 |
| Methods – Bias | 9 | Describe measures taken to address potential sources of bias. | Methods: MRI assessment, p. 6; Discussion: Strengths and Limitations, pp. 13–14 |
| Methods – Study size | 10 | Explain how the study size was determined. | Methods: Study population, p. 5 — all eligible patients during the study period were included; no a priori sample-size calculation |
| Methods – Quantitative variables | 11 | Explain how quantitative variables were handled and, where applicable, why categories were used. | Methods: MRI assessment and outcome variables, Covariates, Statistical analysis, pp. 6–8 |
| Methods – Statistical methods | 12a | Describe statistical methods, including methods used to control for confounding. | Methods: Statistical analysis, pp. 7–8 |
| Methods – Statistical methods | 12b | Describe methods used to examine subgroups and interactions, if applicable. | Methods: Statistical analysis, pp. 7–8 — dose-response model restricted to injected patients |
| Methods – Statistical methods | 12c | Explain how missing data were addressed. | Methods: Study population, p. 5 — missing key exposure or outcome variables were exclusion criteria |
| Methods – Statistical methods | 12d | If relevant, describe analytical methods accounting for the sampling strategy. | Not applicable — no complex sampling strategy |
| Methods – Statistical methods | 12e | Describe any sensitivity analyses. | Not applicable — no sensitivity analyses reported |
| Results – Participants | 13a | Report numbers of individuals included at relevant stages of the study. | Results: Study population, p. 10 — final analytic cohort n = 147 |
| Results – Participants | 13b | Give reasons for non-participation or exclusion at each stage, where available. | Methods: Study population, p. 5 — exclusion criteria reported; stage-specific exclusion counts not reported |
| Results – Participants | 13c | Consider use of a flow diagram. | Not provided; final cohort and eligibility criteria are reported in text |
| Results – Descriptive data | 14a | Report participant characteristics and relevant exposure/confounder information. | Table 1, pp. 8–9; Results: Study population, p. 10 |
| Results – Descriptive data | 14b | Indicate the number of participants with missing data for each variable of interest. | Methods: Study population, p. 5 — cases missing key exposure or outcome variables were excluded |
| Results – Outcome data | 15 | Report numbers or summary measures for outcome variables. | Table 1, pp. 8–9; Results: Walz-based and diameter-based MRI tendon damage, pp. 10–11 |
| Results – Main results | 16a | Provide estimates and precision; make clear which estimates are adjusted and which confounders were included. | Abstract, p. 2; Results, pp. 10–11. Adjusted ORs/ratios or mean differences with 95% CIs are reported; covariates are specified. Unadjusted descriptive group data are shown in Table 1; separate crude regression estimates are not reported. |
| Results – Main results | 16b | Report category boundaries when continuous variables are categorized. | Methods: Covariates, pp. 6–7; occupational workload categorized as low/moderate/high based on documented occupation and expected upper-extremity demands |
| Results – Main results | 16c | If relevant, translate relative risk estimates into absolute risk over a meaningful time period. | Not applicable to this cross-sectional analysis |
| Results – Other analyses | 17 | Report other analyses performed, such as subgroup or dose-response analyses. | Results: Walz-based MRI tendon damage, p. 10 — dose-response analysis among injected patients |
| Discussion – Key results | 18 | Summarize key results with reference to the study objectives. | Discussion, pp. 11–13 |
| Discussion – Limitations | 19 | Discuss study limitations, including potential bias or imprecision and their likely direction/magnitude where possible. | Discussion: Strengths and Limitations, pp. 13–14 |
| Discussion – Interpretation | 20 | Provide a cautious overall interpretation considering objectives, limitations, multiplicity, and other evidence. | Discussion, pp. 11–14; Conclusions, pp. 14–15 |
| Discussion – Generalizability | 21 | Discuss the generalizability/external validity of the findings. | Discussion: Strengths and Limitations, p. 14 |
| Other information – Funding | 22 | State the source and role of funding for the present study. | Declarations: Funding, p. 16 — no funding |

**Supplementary File 1**

STROBE Checklist for Cross-Sectional Studies.

Title: *MRI severity of common extensor origin damage associated with prior glucocorticoid injection in chronic lateral epicondylitis: a retrospective multicenter analysis.*
